# The Lid Domain in Lipases: Structural and Functional Determinant of Enzymatic Properties

**DOI:** 10.3389/fbioe.2017.00016

**Published:** 2017-03-09

**Authors:** Faez Iqbal Khan, Dongming Lan, Rabia Durrani, Weiqian Huan, Zexin Zhao, Yonghua Wang

**Affiliations:** ^1^School of Food Science and Engineering, South China University of Technology, Guangzhou, China; ^2^School of Chemistry and Chemical Engineering, Henan University of Technology, Zhengzhou, China; ^3^School of Bioscience and Bioengineering, South China University of Technology, Guangzhou, China

**Keywords:** lipase, lid domain, thermostability, interfacial activation, protein engineering

## Abstract

Lipases are important industrial enzymes. Most of the lipases operate at lipid–water interfaces enabled by a mobile lid domain located over the active site. Lid protects the active site and hence responsible for catalytic activity. In pure aqueous media, the lid is predominantly closed, whereas in the presence of a hydrophobic layer, it is partially opened. Hence, the lid controls the enzyme activity. In the present review, we have classified lipases into different groups based on the structure of lid domains. It has been observed that thermostable lipases contain larger lid domains with two or more helices, whereas mesophilic lipases tend to have smaller lids in the form of a loop or a helix. Recent developments in lipase engineering addressing the lid regions are critically reviewed here. After on, the dramatic changes in substrate selectivity, activity, and thermostability have been reported. Furthermore, improved computational models can now rationalize these observations by relating it to the mobility of the lid domain. In this contribution, we summarized and critically evaluated the most recent developments in experimental and computational research on lipase lids.

## Introduction

Lipases (triacylglycerol ester hydrolases EC 3.1.1.3) are among the most important industrial enzymes due to their specificity in hydrolysis, interesterification, alcoholysis, acidolysis, esterification, and aminolysis. These enzymes are generally used in different chemical sectors such as detergents, food, bioenergy, flavors, pharmaceuticals, and enantiopure esters and amino acid derivatives used in fine chemicals and agrochemicals (Hasan et al., 2006).

**Figure 1:**
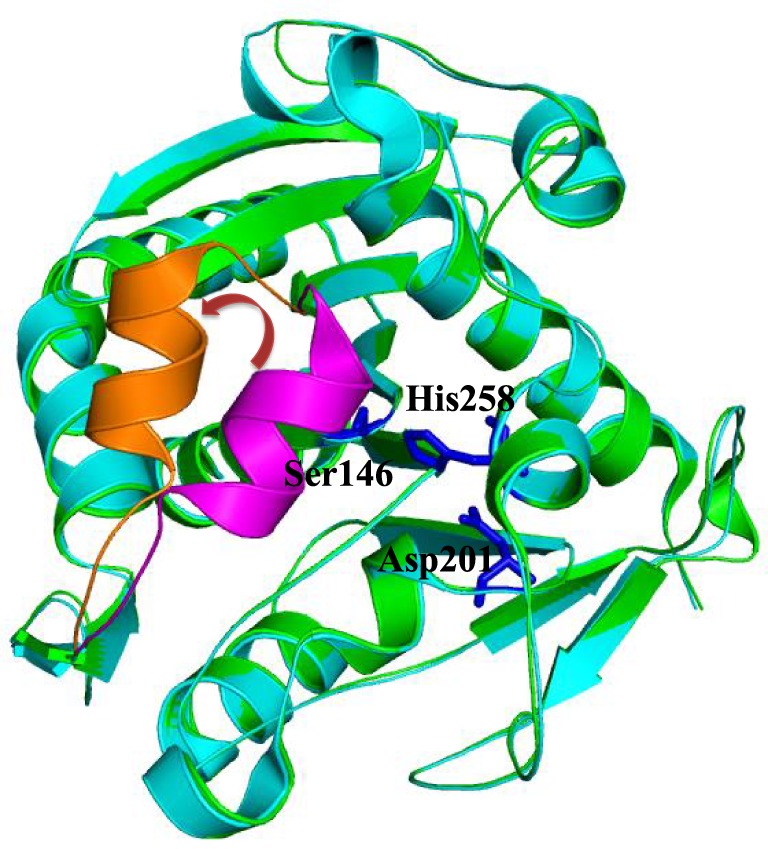
**Superimposition of close (green, PDB code: 1DT3) and open (cyan, PDB code: 1EIN) conformations of *Thermomyces lanuginosus* lipase**. The close lid, open lid, and catalytic triads were highlighted by magenta, orange, and blue colors, respectively.

Lipases operate at the interface between lipid and water (Reis et al., 2009). The important feature of many lipases is the presence of a mobile subdomain lid or flap located over the active site (Brocca et al., 2003). If the lid is closed, the active site is protected from the environment and inaccessible to the substrates, hence the lipase is inactive. In an open conformation, substrates can enter the lipases’ active sites and be converted. In other words, only “open” lipases display catalytic activity (Barbe et al., 2009). For example, the structure of lipase from *Thermomyces lanuginosus* has been resolved both in its closed conformation (PDB code: 1DT3) as well as in its open conformation (PDB code: 1EIN). A comparison of both conformations is shown in Figure 1. In accordance with the generally very low catalytic activity of lipases in mainly aqueous media, it may be assumed that the “closed” conformation prevails under these conditions. Contrarily, in more hydrophobic (organic) media or in the presence of an organic–aqueous interphase, the “open” form may be assumed to be the predominant structure, also in accordance with the generally higher activity of lipases. The increased activity of lipases in the presence of apolar–aqueous interphases is known as “interfacial activation” (Reis et al., 2009).

Lids of lipases are amphipathic structures; in the closed conformation, their hydrophilic side faces the solvent, while the hydrophobic side is directed toward the catalytic pocket (Brocca et al., 2003). As the enzyme shifts to the open conformation, the hydrophobic face becomes exposed and contributes to the substrate-binding region (Yang and Lowe, 2000). Therefore, not only the amphipathic nature of the lid but also its specific amino acid sequence is important for activity and specificity of lipases (Holmquist et al., 1995).

It is known that lipid hydrolysis by lipase is activated by an oil–water interface (Maruyama et al., 2000). X-ray crystallographic analysis has showed that opening of the lid might occur during oil–water interfacial activation, hence allowing substrates access to the active site (Brzozowski et al., 1991).

Most lipases have a lid domain that covers its catalytic triad and the movement of their α-helical lid by rotating around two hinge regions at the lipid–water interface creates a large hydrophobic patch around the catalytic triad, resulting in activation of the lipase (Derewenda et al., 1992; Berg et al., 1998; Cajal et al., 2000).

Nowadays, there are many microorganism genomes that have been sequenced and millions of released raw sequences data have been deposited in database, such as Genbank, DDBJ, and EMBL. Genomic mining by combining bioinformatics analysis and functional screening provides opportunities to find out novel biocatalysts with desired properties for industry application, such as lipases (Masuch et al., 2015; Ufarte et al., 2015). Further modification of the catalytic behaviors of lipase can be achieved by engineering its lid domain. Therefore, the current review article focuses on the classification, mechanism, function, protein engineering, and computational analysis of lid domain of lipases. The following sections will explore the detailed study of lid domain of lipases.

## Classification of Lids

**Figure 2:**
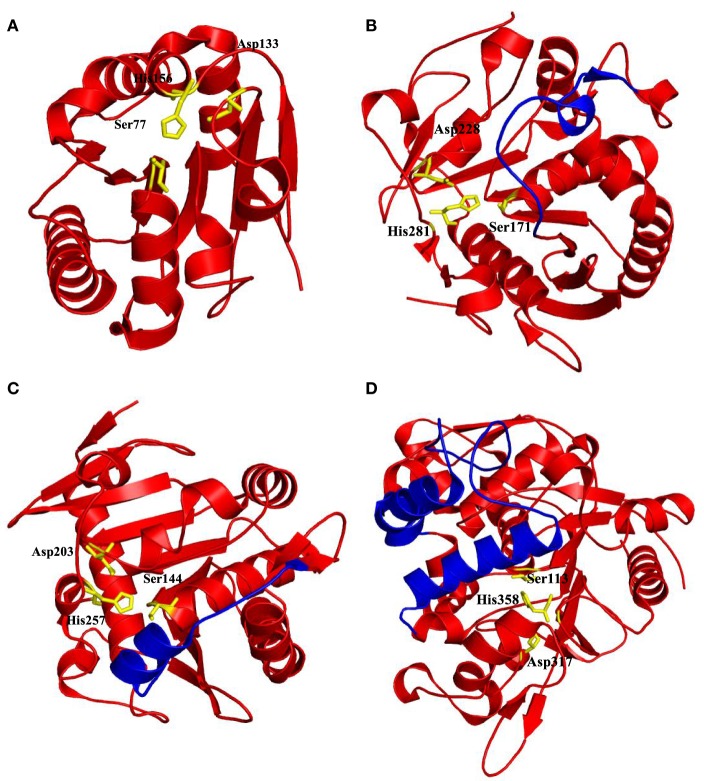
**Some of the lipases shown with the position of catalytic triads residues and lid domains**. **(A)** *Bacillus subtilis* Lipase A with no lid domain (PDB code: 1I6W) (van Pouderoyen et al., 2001), **(B)** *Malassezia globosa* lipase “SMG1” with a lid domain Thr101-Asp119 contains loop form of lid (PDB code: 3UUE) (Xu et al., 2012), **(C)** *Rhizomucor miehei* lipase with a helix lid (PDB code: 3TGL) (Brzozowski et al., 1992), and **(D)** *Geobacillus zalihae* lipase with two helices in its lid domain (PDB code: 2DSN) (Matsumura et al., 2008). Catalytic triad residues were highlighted by yellow sticks and lid domains were highlighted by blue color, respectively.

Lipases can be classified into different groups based on the similarity of sequence, structure, and function. Arpigny and Jaeger (1999) suggested that bacterial lipases can be classified into eight classes according to their conserved amino acid sequences and biochemical properties. Fischer and Pleiss (2003) have generated a lipase engineered database for analyzing sequence–structure–function relationship of α/β-hydrolase fold enzymes, and they proposed a classification of the lipolytic enzymes into GX- and GGGX-hydrolases groups based on the composition of their oxyanion hole. Kourist et al. (2010b) employed 3DM, a commercial structure-based sequence alignment and analysis tool, to analyze 1,172 structurally relative α/β-hydrolase fold enzymes, suggesting that the α/β-hydrolase fold enzyme superfamily can be divided into six families in term of their composition of the catalytic elbow. In this review, we have collected 149 structures of 44 different lipases from the Protein Data Bank (http://www.rcsb.org/). Of which, 25 lipases belong to eukaryotes, and 19 lipases to prokaryotes. Based on the type of lid domain, we have classified these lipases into three groups such as lipases without lids (Table 1), lipases with one loop or one helix lids (Table 2), and lipases with two or more helixes lids (Table 3). A structural comparison of lipases devoid of lids and with lids composed of one or two helices is shown in Figure 2. The position of the lid domain in the structure and the optimum reaction temperature were also summarized. It has been observed that high temperature lipases contain larger lid domains with two or more helices, and all mono- and diacylglycerol lipases have a small lid in the form of a loop or a helix (Table 4).

**Table 1:** **The structure of lipases without lid present in the Protein Data Bank**.

Organisms	PDB code	Reference	Optimum temperature (°C)	Optimum pH
*Bacillus subtilis*	1I6W	Pouderoyen et al. (2001)	35 (Lesuisse et al., 1993)	10 (Lesuisse et al., 1993)
	1ISP	Kawasaki et al. (2002)		
	1R4Z, 1R50	Droge et al. (2006)		
	1T2N, 1T4M	Acharya et al. (2004)		
	2QXT, 2QXU	Rajakumara et al. (2008)		
	3D2A, 3D2B, 3D2C	Ahmad et al. (2008)		
	3QMM	Kamal et al. (2011)		
	3QZU	Augustyniak et al. (2012)		
	5CRI, 5CT4, 5CT5, 5CT6, 5CT8, 5CT9, 5CTA, 5CUR	Nordwald et al. (2015)		
*Streptomyces exfoliatus*	1JFR	Wei et al. (1998)	–	–
*Pseudomonas mendocina*	2FX5	–	–	–
*Candida Antarctica*	1LBS, 1LBT	Uppenberg et al. (1996)	45 (Eom et al., 2013)	7 (Eom et al., 2013)
	1TCA, 1TCB, 1TCC	Uppenberg et al. (1994)		
	3ICV, 3IVW	Qian et al. (2009)		
	3W9B	–		
	4K5Q, 4K6G, 4K6H, 4K6K	Xie et al. (2014)		
	4ZV7	Strzelczyk et al. (2016)		
	5A6V5A71	Benjamin et al. (2015)		
*Cavia porcellus*	1GPL	Withersmartinez et al. (1996)	–	–

**Table 2:** **The structures of lipases with a loop or helical lid present in the Protein Data Bank**.

Organism	PDB code	Reference	Lid	Optimum temperature (°C)	Optimum pH
*Bacillus* sp. (strain H-257)	3RLI, 3RM3	Rengachari et al. (2012)	119I-164T	75 (Imamura and Kitaura, 2000)	6–8 (Imamura and Kitaura, 2000)
	4KE6, 4KE7, 4KE8, 4KE9, 4KEA	Rengachari et al. (2013)			

*Malassezia globosa*	3UUE, 3UUF	Xu et al. (2012)	99E-116W	25 (Zisis et al., 2015)	6 (Zisis et al., 2015)
	4ZRD, 4ZRE	Guo et al. (2015)			

*Bos taurus*	1AKN, 1AQL	Wang et al. (1997)	116G-129E	–	–
	2BCE	Chen et al. (1998)			

*Homo sapiens* (bile salt-activated lipase)	1F6W	Terzyan et al. (2000)	115H-125Y	–	–
	1JMY	Moore et al. (2001)			

*Burkholderia cepacia*	1HQD	Nardini et al. (2000)	130D-158Q	45 (Rathi et al., 2001)	6 (Rathi et al., 2001)
	1OIL	Luic et al. (2001)			
	1YS1, 1YS2	Kim et al. (1997)			
	2LIP, 3LIP	Mezzetti et al. (2005)			
	2NW6	Schomburg et al. (1997)			
	4LIP, 5LIP	Luic´ et al. (2008)			

*Acinetobacter baumannii*	4OPM	–	178T-195K	–	–

*Photobacterium* sp. M37	2ORY	Jung et al. (2008)	91G-104D	–	–

*Thermomyces lanuginosus*	1DT3, 1DT5, 1DTE, 1DU4, 1EIN	Brzozowski et al. (2000)	81R-96D	35 (Fernandes et al., 2004)	8 (Fernandes et al., 2004)
	1GT6	Yapoudjian et al. (2002)			
	1TIB, 1TIC	Derewenda et al. (1994b)			
	4DYH, 4EA6, 4FLF, 4GBG, 4GHW, 4GI1, 4GLB, 4GWL, 4KJX, 4N8S, 4S0X, 4ZGB	–			

*Gibberella zeae*	3NGM	Derewenda et al. (1994b)	80R-90D	35 (Long et al., 2010)	7 (Long et al., 2010)

*Rhizomucor miehei*	1TGL	Brady et al. (1990)	80R-95V	45 (Huang et al., 2014)	8 (Huang et al., 2014)
	3TGL	Brzozowski et al. (1992)			
	4TGL	Derewenda et al. (1992)			
	5TGL	Brzozowski et al. (1991)			

*Rhizopus niveus*	1LGY	Kohno et al. (1996)	81R-95F	35–40 (Kohno et al., 1994)	6–6.5 (Kohno et al., 1994)

*Candida cylindracea*	1LLF	Pletnev et al. (2003)	66E-92P		

*Yarrowia lipolytica*	3O0D	Bordes et al. (2010)	88T-105L	37 (Corzo and Revah, 1999)	6 (Corzo and Revah, 1999)

*Penicillium camemberti*	1TIA	Derewenda et al. (1994a)	82G-96V	40 (Isobe et al., 1992)	6 (Isobe et al., 1992)

*Arabidopsis thaliana*	2YIJ	–	154R-169G	30 (Kim et al., 2011)	6.5 (Kim et al., 2011)

*Burkholderia glumae*	1CVL	Lang et al. (1996)	130D-156T	–	–
	1TAH	Noble et al. (1993)			
	2ES4	Pauwels et al. (2006)			
	1QGE	–			

*Serratia marcescens*	2QUA, 2QUB	Meier et al. (2007)	141R-169K	–	–

*H. sapiens* (human monoglyceride lipase)	3HJU	Labar et al. (2010)	156A-172P	–	–
	3JW8, 3JWE	Bertrand et al. (2009)			
	3PE6	Schalk-Hihi et al. (2011)			
	4UUQ	Griebel et al. (2015)			

**Table 3:** **The structures of lipases with multiple helical lid present in the Protein Data Bank**.

Organism	PDB code	Reference	Lid	Optimum temperature (°C)	Optimum pH
*Proteus mirabilis*	4GW3, 4GXN	Korman and Ju (2012)	121K-160L	35 (Gao et al., 2009)	9 (Gao et al., 2009)

*Pseudomonas aeruginosa*	1EX9	Nardini et al. (2000)	122P-163N	50 (Gilbert et al., 1991)	8.5–8 (Gilbert et al., 1991)

*Pseudomonas* sp. MIS38	2Z8X, 2Z8Z	Angkawidjaja et al. (2007)	45F-74P	–	–
	2ZJ6, 2ZJ7	Kuwahara et al. (2008)	146P-167G		
	2ZVD, 3A6Z, 3A70	Angkawidjaja et al. (2010)			

*Geobacillus stearothermophilus*	1KU0	Jeong et al. (2002)	173M-238D	60–65 (Kim et al., 1998)	9 (Kim et al., 1998)

*G. stearothermophilus*	1JI3	Safra et al. (2002)	173M-238D	65 (Kim et al., 2000)	9 (Kim et al., 2000)

*Geobacillus zalihae*	2DSN	Matsumura et al. (2008)	173M-238D	70 (Schmidt-Dannert et al., 1997)	9 (Schmidt-Dannert et al., 1997)

*Geobacillus thermocatenulatus*	2W22	Carrascolópez et al. (2009)	174M-239D	50 (Schmidt-Dannert et al., 1996)	9 (Schmidt-Dannert et al., 1996)

*Staphylococcus hyicus*	2HIH	Tiesinga et al. (2007)	185D-240D	37 (Schmidt-Dannert et al., 1996)	8.5 (Schmidt-Dannert et al., 1996)

Uncultured bacterium	3FAK	Nam et al. (2009b)	1M-36V	35 (Nam et al., 2009b)	5 (Nam et al., 2009b)

Uncultured Bacterium	3DNM	Nam et al. (2009a)	16M-49C	40 (Nam et al., 2009a)	5 (Nam et al., 2009a)
	3K6K		193S-223E		

*Archaeoglobus fulgidus*	2ZYH, 2ZYI, 2ZYR, 2ZYS	Chen et al. (2009)	62T-101K	70–90 (Chen et al., 2009)	10–11 (Chen et al., 2009)

*Geotrichum candidum*	1THG	Schrag and Cygler (1993)	61C-105C		

*Candida rugosa*	1CRL	Grochulski et al. (1993)	60C-97C	30 (Korbekandi et al., 2008)	7 (Korbekandi et al., 2008)
	1LPM, 1LPS	Cygler et al. (1994)			
	1LPN, 1LPO, 1LPP	Grochulski et al. (1994)			
	1TRH	Pawel et al. (2008)			
	3RAR	Colton et al. (2011)			

*Candida antarctica*	2VEO	Ericsson et al. (2008)	217S-308E	50–70 (Pfeffer et al., 2006)	7 (Pfeffer et al., 2006)
	3GUU				

*Homo sapiens*	1HLG	Roussel et al. (1999)	209D-251F	–	–

*Canis lupus*	1K8Q	Roussel et al. (2002)	208G-251L	–	–

*Sus scrofa*	1ETH	Hermoso et al. (1996)	238C-262C	–	–

*C. lupus*	1RP1	Roussel et al. (1998a)	237C-261C	–	–

*Rattus norvegicus*	1BU8	Roussel et al. (1998b)	237C-261C	–	–

*H. sapiens*	1N8S	Tilbeurgh et al. (1992)	237C-261C	–	–
	1LPA, 1LPB	Van et al. (1993)			

*Equus caballus*	1HPL	Bourne et al. (1994)	237C-261C	–	–

**Table 4:** **The list of lipases with some special substrate selectivity**.

Organism	PDB code	Substrate specificity	Lid type
*Bacillus* sp. (strain H-257)	3RM3	Monoacylglycerol lipase	Loop
*Malassezia globosa*	3UUF	Mono- and diacylglycerol lipase	Loop
*Penicillium camemberti*	1TIA	Mono- and diacylglycerol lipase	One helix
*Arabidopsis thaliana*	2YIJ	Mono- and diacylglycerol lipase	One helix
*Homo sapiens*	3HJU	Monoacylglycerol lipase	One helix

## Interfacial Activation

The lipolytic activity of some lipases significantly increases beyond the critical micellar concentration of substrate. This “interfacial activation” phenomenon generally ascribes to the presence of an amphiphilic lid structure, which undergoes conformational changes in contact with the micellar substrates (Cambillau et al., 1996). The movement of the lid in lipases structure with substrate analogs has been also found (Brzozowski et al., 1991), which provides structural evidence for this phenomenon. Further, Cheng et al. (2012) reported that lipase from *Pseudomonas* sp. MIS38 (PML) does not undergo interfacial activation after deletion of its lid2, and they proposed that lid2 is important for interfacial activation of PML. However, lipases with mini- or without lid domains such as guinea pig and lipase B from *Candida antarctica* are found not to show any interfacial activation (Hjorth et al., 1993; Martinelle et al., 1995). It is surprising that coypu lipase containing a 23-amino acid lid domain did not exhibit interfacial activation (Thirstrup et al., 1994). On the basis of these observations, Verger (1997) suggested that interfacial activation and the existence of lid domain are not suitable criteria to determine a lipolytic enzyme as a lipase.

## Effects on Activity and Substrate Specificity

The vital role of the lipase lid domain in substrate selectivity and activity has been confirmed by several approaches such as lid swapping and site-directed mutagenesis. Dugi et al. (1995) constructed chimeras of hepatic lipase (HL) with lipoprotein lipase (LPL) lid, and LPL with HL lid to analyze their activity with triacylglycrerols and phospholipid as substrate. Chimeric LPL that contains the lid of HL had reduced triacylglycrerol hydrolyzing activity, but increased phospholipase activity. In contrast, chimeric HL that contains the LPL lid was found to be more active against triacylglycrerols and less active against phospholipid substrate. This study clearly showed that the triglyceride and phospholipid hydrolysis activity of lipase can be alerted by swapping the lids of LPL and HL.

Accordingly, Brocca et al. (2003) generated a *Candida rugosa* lipase (CRL) 1 mutant with lid domain of CRL3. This CRL1 mutant displayed 200-fold higher activity toward cholesterol esters, showing that the lid was involved in determining the cholesterol esterase activity of CRL. Santarossa et al. (2005) performed site-directed substitution of residual T137 and T138 in lid domain of *Pseudomonas fragi* lipase with the valine and asparagines, respectively. The mutants showed a different chain length preference profile as compared to the wild-type lipase. The lipase activity can be modulated by mutagenesis in the lid domain. Substitution of serine 154 and glycine 152 in lid of *Pseudomonas* sp. CR611 Lip I.3 lipase with threonine and leucine resulting in fivefold and twofold increase in activity on 4-methylumbelliferyl-heptanoate, respectively (Panizza et al., 2015). Similarly, *Bacillus thermocatenulatus* lipase activity was increased up to 2.6-fold by substitution of F181 with alanine due to decrease in steric hindrance in the lid domain (Karkhane et al., 2009). A detailed analysis of shape and physicochemical properties of lipase-binding sites were analyzed by Pleiss et al. (1998) in order to understand the molecular basis of substrate specificity. Overall, these studies confirm the pivotal role of lids in selectivity and activity of lipases.

## Effects on Thermostability

Next to selectivity and activity, thermostability is one of the most desirable traits of lipases (Dizge et al., 2009; Avila-Cisneros et al., 2014; Khan et al., 2016). Several factors define this property such as the number of hydrogen bonds, salt bridges, stabilization of secondary structures, occurrence of disulfide bonds, higher number of proline residues, higher polar surface area, shortening of loops, and stabilization of the lid domain (Pack and Yoo, 2004; Santarossa et al., 2005; Zhou et al., 2008; Khan et al., 2015a,b).

It has been found that the activity and thermostability of lipases can be altered by modifications in their lid domains. Timucin and Sezerman (2013) found that the conserved tryptophan of the lid region potentiates the thermostability and thermoactivity in bacterial thermoalkalophilic lipases from *B. thermocatenulatus* that stabilizes the aggregates by forming new intermolecular interactions. Yang et al. (2015) characterized the thermostable lipase from *Pseudomonas* sp. R0-14 and found that, when the lid is in the open conformation, the proportion of α-helices increased. An increase in the number of α-helices may make the lipase more thermostable in open conformation. Dror et al. (2014) employed protein engineering to enhance the stability of *Geobacillus stearothermophilus* Lipase T6 in methanol. They found that Gln185 situated on the lipase α-helix lid has an important role in the lipase interfacial activation. The substitution of Gln185 to Leu resulted in an improved stability in organic solvents due to the replacement of the polar glutamine by the more hydrophobic leucine. This hydrophobization also improved the structural stability of the enzyme by facilitating the interaction between the solvent molecules and the lid surface.

The substitutions of amino acids in the lid region of *R. chinensis* lipase affect not only its substrate specificity but also its thermostability. Probably, this is due to destabilization of lid structure by disrupting the H-bond interaction in the lid region (Zhu et al., 2013). Yu et al. (2012) demonstrated that introducing a disulfide bond in the lid hinge region of *R. chinensis* lipase increases thermostability and alters the acyl chain length specificity due to stabilization of the geometric structure of the lid region. Wu et al. (2010) suggested that the conserved residue Tyr224 of *Geobacillus* sp. RD-2 lipase is very close to the lid domain and is the key amino acid residue, which determines the thermostability of lipase. Santarossa et al. (2005) found that the mutations in the lid region of *P. fragi* lipase effect the chain length specificity and thermostability. The above studies concluded that the lid region not only plays an important role in the function of the lipase but also stabilizes the helix.

It has been also found that substitutions such as Val72Gly and Val72Ala causes higher activity and enantioselectivity of *Penicillium expansum* lipase, but decreases the thermostability (Tang et al., 2013). The substitution of Asp189 residue in the lid domain of *Geobacillus* sp. NTU 03 lipase also leads to a loss in its thermostability but exhibited higher activity (Shih and Pan, 2011). Sheng et al. (2014) employed the circular permutation protein engineering technique to acquire active mutants of *Yarrowia lipolytica* lipase. They also found that most of the functional mutations are seen in the surface-exposed loop region in close proximity to the lid domain, which implies the steric effect of the lid on lipase activity and substrate specificity, but there were no change in thermostability. So, the change in amino acid residues of lid region may lead to increase as well as decrease in stability depending upon nature of amino acid substitution.

## Engineering the Lid Domains of Lipases

Most lipases bear a flexible lid close to the active site. This dynamic domain is very likely to affect both stability as well as catalytic properties of the biocatalyst and is, therefore, an attractive target for protein design (Kourist et al., 2010a). For instance, the modification of the lid region by site-directed mutagenesis of lid domain or hinge region or by lid swapping (Table 5), resulted in changes in the substrate specificity (Yu et al., 2014), enantioselectivity (Secundo et al., 2004; Gao et al., 2011), and stability (Yu et al., 2012) toward detergents (Brocca et al., 2003) and organic solvents (Secundo et al., 2004), which could turn into lack of oil–water interfacial activation (Shu et al., 2011; Tang et al., 2015).

**Table 5:** **Properties of lipase variants generated by lid and hinge region modification**.

Enzyme	Mutants	Mutants description	Mutants property	Reference
*Proteus* sp. LipK107	E130L + K131I	The hydrophobicity of the lid domain increases	The eep (%) and E on the resolution of racemic 1-phenylethanol increased by 1.36 and 137.6%, respectively	Gao et al. (2011)
	T138V		The eep (%) and E on the resolution of racemic 1-phenylethanol increased by 0.52 and 30.6%, respectively	

*Rhizopus chinensis* lipase S4-3	S4-3M	The lid of S4-3 was swapped with ferulic acid esterase from *Aspergillus niger*	Specific activity toward short-chain substrates increased by 7.2-fold (C3) and 38.0-fold (C2), respectively	Yu et al. (2014)
	S4-3N	The lid of S4-3 was swapped with *Rhizomucor miehei* lipase	Specific activity toward substrates (C2, C6, C8, C12, and C16) increased by 1.5- to 3.3-fold and reduced 40% toward tristearin (C18)	

*Candida rugosa* lipase (CRL) Trx-LIP4	CRL4LID1	The lid of CRL4 was swapped with CRL1	Hydrolytic activity decreased by 85%, changed CLP, and reduced enantioselectivity	Akoh et al. (2004)

CRL LIP1	CRL1LID3	The lid of CRL1 was swapped with CRL3	Specific activity toward cholesterol esters increased by 200-fold, enantioselectivity and activity reduced in organic solvent	Akoh et al. (2004)

*Candida antarctica* lipase B (CALB)	CALB-*N. crassa*	The lid of CALB was swapped with CALB homolog from *Neurospora crassa* lipase	Hydrolytic activity increased on simple esters, specifically, substrates with Ca branching on the carboxylic side, and increased enantioselectivity in hydrolysis of racemic ethyl 2-phenylpropanoate (E > 50)	Skjot et al. (2009)
	CALB-*G. zeae*	The lid of CALB was swapped with CALB homologs from *Gibberella zeae* lipase		

*Penicillium expansum* lipase (PEL)	T66L + D70N	The mutant residues are located at the lid (D70N) and the lid hinge region (T66L, E83K) of PEL	Specific activity toward *p*-nitrophenyl palmitate increased by 136.4-fold	Tang et al. (2015)
	E83K		Specific activity toward *p*-nitrophenyl butyrate increased by 136.4-fold, but lack interfacial activation	

*A. niger* lipase (ANL)	S84G	The mutant residues are located at the lid hinge region of ANL	Specific activity toward *p*-nitrophenyl esters decreased and displayed a pronounced interfacial activation	Shu et al. (2011)
	D99P		Specific activity toward *p*-nitrophenyl palmitate increased by 2.2-fold and displayed no interfacial activation	

*R. chinensis* lipase	F95C + F214C	A disulfide bridge was introduced into the lipase from *R. chinensis* in the hinge region of the lid	The half-life t1/2 value increased by 11-fold at 60°C and the Tm increase by 7°C, but the catalytic efficiency toward pNPP decreased by 1.5-fold	Yu et al. (2012)

### Site-Directed Mutagenesis of the Lid Domains

Site-directed mutagenesis of the lid domain of *Proteus* sp. LipK107 lipase improved the conversion of 1-phenylethanol with a slight increase in enantiodiscrimination (Gao et al., 2011). Increase in the hydrophobicity of the lid in case of mutants Glu130Leu + Lys131Ile and Thr138Val resulted in higher conversions of 1-phenylethanol than LipK107. On the contrary, the mutant Ile128Glu + Val129Asp has lower conversions than that of LipK107, and the E value (enantiomeric ratio) of the resolution changed in accordance with the conversions. Several studies were carried out to understand the mechanism of lid-opening and closing. It has been shown that site-directed mutagenesis of the lid region in *T. lanuginosus* lipase could possibly generate lipase variants with attractive features such as high lipase activity, fast activation at the lipid interface, ability to act on water soluble substrates, and enhanced calcium independence (Skjold-Jorgensen et al., 2014). Skjold-Jorgensen et al. (2017) studied controlled lid-opening in *T. lanuginosus* lipase by introducing disulfide bond between C86 and C255 residues that causes strained closure of the lid-domain. The formation of disulfide bind leads to locking of lid in a closed conformation. Upon unlocking, enzymatic activity was fully restored. They showed that this intrinsic bond enables control of both lipase activity and interfacial binding. They also suggested the key role that the lid plays in determining the polarity-dependent activation of lipases using a combination of methods measuring enzymatic activity, detecting structural changes using the tryptophan-induced quenching method, and calculating the lid opening energies using an MD simulations, and suggested that mutagenesis of the lid can lower the energy barrier associated with lid opening (Skjold-Jorgensen et al., 2016). Tryptophan-induced quenching fluorescence method has been applied to successfully measure the lid movements in *T. lanuginosus* lipase and its variants in solvents with different dielectric constants (Skjold-Jorgensen et al., 2015). The results indicated that lid movement is highly dependent on the particular lid residue composition as well as solvent polarity. In other words, lipases are more active in low polarity solvents because the lid adopts an open conformation, and relatively small conformational changes in the lid region play a key role in the activation mechanism.

### Lid Swapping

An interesting approach for the protein engineering of lipases is the exchange of lids of homologous enzymes, also referred to as “lid swapping.” The amphipathic nature of the lid is very important for the substrate specificity, and it provides new insight into the structural basis of lipase substrate specificity and a way to tune the substrate preference of lipases.

The substrate specificity of *R. chinensis* lipase S4-3 was successfully modified by replacing the hydrophobic lid (85.7% polar residues) with a hydrophilic lid (57.1% polar residues) of ferulic acid esterase from *Aspergillus niger* (AnFaeA) or a hydrophobic lid of *Rhizomucor miehei* lipase (RML) (Yu et al., 2014). The most apparent changes by lid swapping were that the replacement of the S4-3 lid with that of AnFaeA shifted the specificity toward short-chain substrates (C2–C6) compared with that of the parent (C12), increased by 7.2-fold (C3) and 38.0-fold (C2), respectively. While the replacement of the S4-3 lid with that of RML caused a 1.5- to 3.3-fold increase in the specific activity toward those substrates (C2, C6, C8, C12, and C16) and a 40% reduction toward tristearin (C18) compared with the corresponding activity of the parent.

In one of the study, novel *Candida antarctica* lipase B (CALB) mutants in which the entire CALB lid region is substituted with that of homologs (*Neurospora crassa* and *Gibberella zeae*) were characterized (Skjot et al., 2009). It revealed several interesting properties such as increased hydrolytic activity on simple esters and much increased enantioselectivity in hydrolysis of racemic ethyl 2-phenylpropanoate (E > 50). *C. rugosa* LIP4 lipase was also studied by exchanging the lid regions from the other four *C. rugosa* isoforms (LIP1, LIP2, LIP3, and LIP5; and corresponding lids 1, 2, 3, and 5) with that of LIP4, respectively (Akoh et al., 2004). Lid swapping resulted in increased hydrolytic activities toward tributyrin of the chimeric LIP4/lid2 and LIP4/lid3, whereas chimeric LIP4/lid1 and LIP4/lid5 activities decreased, compared with the native LIP4. Furthermore, Brocca et al. (2003) substituted the lid sequences from isoenzymes *C. rugosa* LIP3, which has high activity toward cholesterol esters, to the LIP1, which had little cholesterol esterase activity in its native form. It revealed that the chimeric LIP1/lid3 specific activity toward cholesterol esters increased 200-fold. Secundo et al. (2004) found that the chimeric *C. rugosa* LIP1/lid3 was less active and enantioselective than the wild type for reactions of alcoholysis of chloroethyl-2-hydroxy hexanoate with methanol and of vinyl acetate with 6-methyl-5-hepten-2-ol in organic solvent. They postulated that the decrease in activity may be due to the chimera enzyme having a lower proportion of enzyme molecule in the open form, thereby hindering access to the enzyme active site.

### Site-Directed Mutagenesis of Hinge Region

It is believed that interfacial activation of lipases involves conformational changes of the mobile lid domains. Derewenda et al. (1992) reported that the specific dihedral angles (φ and ψ) conformations of the hinge region amino acids experienced dramatic changes during the process of *R. miehei* interfacial activation. At the N-terminal end of the lid, Ser83 and Ser84 undergo conformational changes. Ser83, in spite of a change in the φ angle of 60° remains within the γR region of the Ramachandran plot, while Ser84 changes its conformation from δ to β region with a change in the ψ angle of 90°.

Shu et al. (2011) found that Asp99Pro and Ly108Glu mutants of *A. niger* lipase (ANL) become oil–water interface independent lipase, probably because of change in the β-sheet configuration of the second hinge region at the side of the lid domain. Three ANL mutants such as ANL-Ser84Gly, ANL-Asp99Pro, and ANL-Lys108Glu were constructed base on the fact that Ser84, Asp99, and Lys108 might be in the hinge region of the lid domain of ANL. ANL-Ser84Gly displayed interfacial activation, while ANL-Asp99Pro and ANL-Lys108Glu displayed no interfacial activation. The specific activity of ANL-Ser84Gly toward *p*-nitrophenyl esters decreases as compared to the wild-type enzyme, while the specific activity of ANL-Asp99Pro increases toward *p*-nitrophenyl palmitate by 2.2-fold.

## Computational Approaches

The behavior of the lid domain and the dynamics of lipase at different temperatures and solvent conditions can be understood by *in silico* methods and computational approaches. The computational methods can help to predict the impact of mutations in the lid of lipase in order to understand the importance of a particular residue. Molecular dynamics (MD) simulations studies can predict the behavior of the lid domain at different temperature, pH and in a particular solvent. Recently, Haque and Prabhu (2016) performed MD simulation of double mutant porcine pancreatic lipase in open and closed conformations using ethanol, toluene, and octanol as solvent to explain the dynamics of lid opening. They found that the Asp250Val and Glu254Leu mutants showed lid opening at higher temperature suggesting the important role of these residues in holding the lid in closed conformation. Also, the dynamics of lid opening was faster in octanol than in water, due to the fact that non-polar solvents favor open conformation of the lid.

Likewise, Jiang et al. (2014) performed a MD simulation study on *Y. lipolytica* lipase in methanol and hexane and proposed a lid closure mechanism. They suggested that the lipase undergoes a greater conformational change in methanol, where several regions such as Ser274-Asn288 and Thr106-His126 were found to interact with the lid region. They proposed that the closure mechanism of the *Y. lipolytica* lipase is due to a double-lid movement in methanol.

*Candida antarctica* lipase B is one of the lipase that displays an enhanced catalytic rate for bulky substrates when adsorbed to a hydrophobic interface. It was proposed that the increased activity of this lipase is due to conformational changes leading to a more open active site. This hypothesis is supported by MD simulations and docking studies suggesting the presence of a highly mobile lid. Molecular docking study confirmed that a highly open conformation is required for binding large, bulky substrates (Zisis et al., 2015). Ganjalikhany et al. (2012) demonstrated the flexibility of the lid region of CALB using comparative MD simulation and essential dynamics analysis carried out at different temperatures, showing that the opening of the lid is temperature dependent. A similar approach was used by Rahman et al. (2012) T1 lipase confirming this temperature-dependency. They found that the lid movement was only observed in the presence of an interface and that the activation process is temperature-dependent. The structural rearrangement of the lid domain was caused by the interaction between the hydrophobic residues of the lid with octane. So, there may be several factors responsible for the mobility of the lid.

Disulfide bonds near the lid region play an important role in stabilization of its helical structure. Recently, Singh et al. (2016) predicted that the disruption of disulfide bonds lower the activation energy and improved catalytic efficiency of *Trichosporon asahii* MSR54 lipase. Using MD simulation methods, they predicted a mutant of this lipase with fourfold increased specific activity with a lower temperature optimum. *In silico* analysis suggested that there are two lids in this lipase and both of them are opened at 40°C through clockwise and anticlockwise rotations, respectively.

The computational analyses were also helpful in understanding the mechanism of thermoactivity and thermostability and the role of conserved tryptophan residue in bacterial thermoalkalophilic lipases (Timucin and Sezerman, 2013). It has been found that residue Trp211 in the lid region stabilized the intermolecular interactions in the dimeric lipase and that it is critical to the stability of the monomeric lipase. Dror et al. (2014) applied *in silico* modeling technique and concluded that the amino acid substitution Gln185Leu facilitates a closed lid conformation, and the enhanced stability of His86Tyr and Ala269Thr mutants was due to formation of new hydrogen bonds in case of *G. stearothermophilus* lipase.

Computational methods are also helpful to change the position of the lid to generate an open conformation of lipase in the absence of crystal structure. Nasr et al. (2013) generated open conformation of monoacylglycerol lipase and performed MD simulations. They suggested that lid region was found to interact with the nanodisc phospholipid bilayer and penetrated into the phospholipid bilayer.

The lipase from *Pseudomonas* sp. MIS38 has two lids, which greatly change its conformation upon substrate binding. Cheng et al. (2012) employed computational approaches to compare the tertiary structures in closed and open conformations. They proposed that a hydrophobic surface is formed by these lids, which is necessary to hold the substrates firmly in the active site. Barbe et al. (2011) applied an advanced computational molecular modeling robotics approach with fully atomistic description to investigate the geometrically feasible transition pathways between *Burkholderia cepacia* lipase lid conformations and classical molecular mechanics to evaluate pathway energetic under the influence of solvent. They proposed a descriptive analysis of intermediate conformations of *B. cepacia* lid. Rehm et al. (2010) performed MD simulations study on different lipases from *C. rugosa, R. miehei*, and *Thermomyces lanuginosa*. The results from MD analysis suggested that in all the three lipases, opening and closing of lids were driven by the solvent and independent of a bound substrate molecule.

## Conclusion and Future Perspectives

The role of the lid on enzyme activity is very complex because it involves specific interactions with substrate molecules and controls the equilibria between active and inactive enzyme conformations. The lid is important for substrate binding as it undergoes dramatic shift that changes the secondary structure of lipase-binding site from closed lid structure to an open structure. We have classified lipases based on the different types of lid domain. Some common and novel characteristics of lipases can be deduced from the nature of lid domain. Lipases that have similar sequence or length of lid could have similar mechanism of action. Different characteristics of lipases including substrate preference, themostablility, and interfacial properties can also be predicted by comparing the lid domain. The lid domain has a close relationship with the substrate specificity of lipases. This makes it a “hot spot” for protein engineering to modulate the lipases catalytic properties that might fulfill the demand of industrial application. Various efforts such as modifications of lid domain using site-directed mutagenesis, lid swapping, introduction of extra bonds, and computational approaches have been employed to modify the activity and thermostability of lipases. Further advancement in the bioinformatics tools will help to predict the accurate function of amino acids present near the lid region of lipases. Protein engineering of lid may provide an opportunity for better understanding of the structural basis of the lipases property. There is a possibility of using these protein engineered thermostable lipases as industrial enzymes at high temperatures.

## Author Contributions

FK and DL wrote the manuscript; FK has drawn the figures, RD, ZZ, and WH prepared the table; YW revised the manuscript.

## Conflict of Interest Statement

The authors declare that the research was conducted in the absence of any commercial or financial relationships that could be construed as a potential conflict of interest. The reviewer SS and handling editor declared their shared affiliation, and the handling editor states that the process nevertheless met the standards of a fair and objective review.
